# Chromosome-scale genome assembly and annotation of *Cotoneaster glaucophyllus*

**DOI:** 10.1038/s41597-024-03246-8

**Published:** 2024-04-22

**Authors:** Kaikai Meng, Wenbo Liao, Shaolong Wei, Sufang Chen, Mingwan Li, Yongpeng Ma, Qiang Fan

**Affiliations:** 1 State Key Laboratory of Biocontrol and Guangdong Provincial Key Laboratory of Plant Resources, School of Life Sciences, Sun Yat-sen University, Guangzhou, 510275 China; 2 Guangxi Key Laboratory of Quality and Safety Control for Subtropical Fruits, Guangxi Subtropical Crops Research Institute, Nanning, 530001 China; 3 College of Forestry, Henan Agricultural University, Zhengzhou, 450002 China; 4 State Key Laboratory of Plant Diversity and Specialty Crops, Kunming Institute of Botany, Chinese Academy of Sciences, Kunming, 650201 China

**Keywords:** Plant evolution, Chromosomes

## Abstract

*Cotoneaster glaucophyllus* is a semi-evergreen plant that blossoms in late summer, producing dense, attractive, fragrant white flowers with significant ornamental and ecological value. Here, a chromosome-scale genome assembly was obtained by integrating PacBio and Illumina sequencing data with the aid of Hi-C technology. The genome assembly was 563.3 Mb in length, with contig N50 and scaffold N50 values of ~6 Mb and ~31 Mb, respectively. Most (95.59%) of the sequences were anchored onto 17 pseudochromosomes (538.4 Mb). We predicted 35,856 protein-coding genes, 1,401 miRNAs, 655 tRNAs, 425 rRNAs, and 795 snRNAs. The functions of 34,967 genes (97.52%) were predicted. The availability of this chromosome-level genome will provide valuable resources for molecular studies of this species, facilitating future research on speciation, functional genomics, and comparative genomics within the Rosaceae family.

## Background & Summary

Species of the genus *Cotoneaster* Medic. belong to the Malinae subtribe of the Rosaceae family^1^, and are primarily distributed in continental Eurasia, with a remarkable species diversity in the biodiversity hotspots of the Himalayas and the Hengduan Mountains (HDM)^2^. Taxonomic difficulties for this genus have been caused by various evolutionary events, including hybridization, polyploidization, and apomixis. A comprehensive phylogenetic analysis of this genus has been conducted using genome-skimming data, but with the genome of *Eriobotrya japonica* serving as the mapping reference^3^, which might introduce mapping errors, incorrect alignments, difficulties in identifying orthologous genes, and genome annotation issues.

Based on morphological characteristics and molecular evidences, two subgenera or sections have been proposed: *Cotoneaster*, characterized by predominantly red or pink flowers with erect petals, and *Chaenopetalum*, noted for its primarily white flowers with spreading petals^2–5^. Notably, only approximately 10% of *Cotoneaster* species are diploid^2^. *Cotoneaster glaucophyllus*, as a representative member of the *Chaenopetalum* subgenus and a diploid species, has a distinct distribution in the southeastern of Hengduan Mountains and on the Yunnan-Guizhou Plateau. It is a semi-evergreen shrub that blossoms in late summer, exhibiting dense, showy, fragrant white flowers, and bears long-lasting fruits in early winter, potentially making it an important ornamental plants^2,6,7^. With continuous advancements in sequencing technology, abundant genome resources for numerous Rosaceae species have been extensively documented^8–12^. However, the lack of whole-genome sequencing in *Cotoneaster* species has been a significant obstacle in further understanding the gene functions, evolutionary history, and conservation of this complicated genus (up to 370 species).

**Fig. 1:**
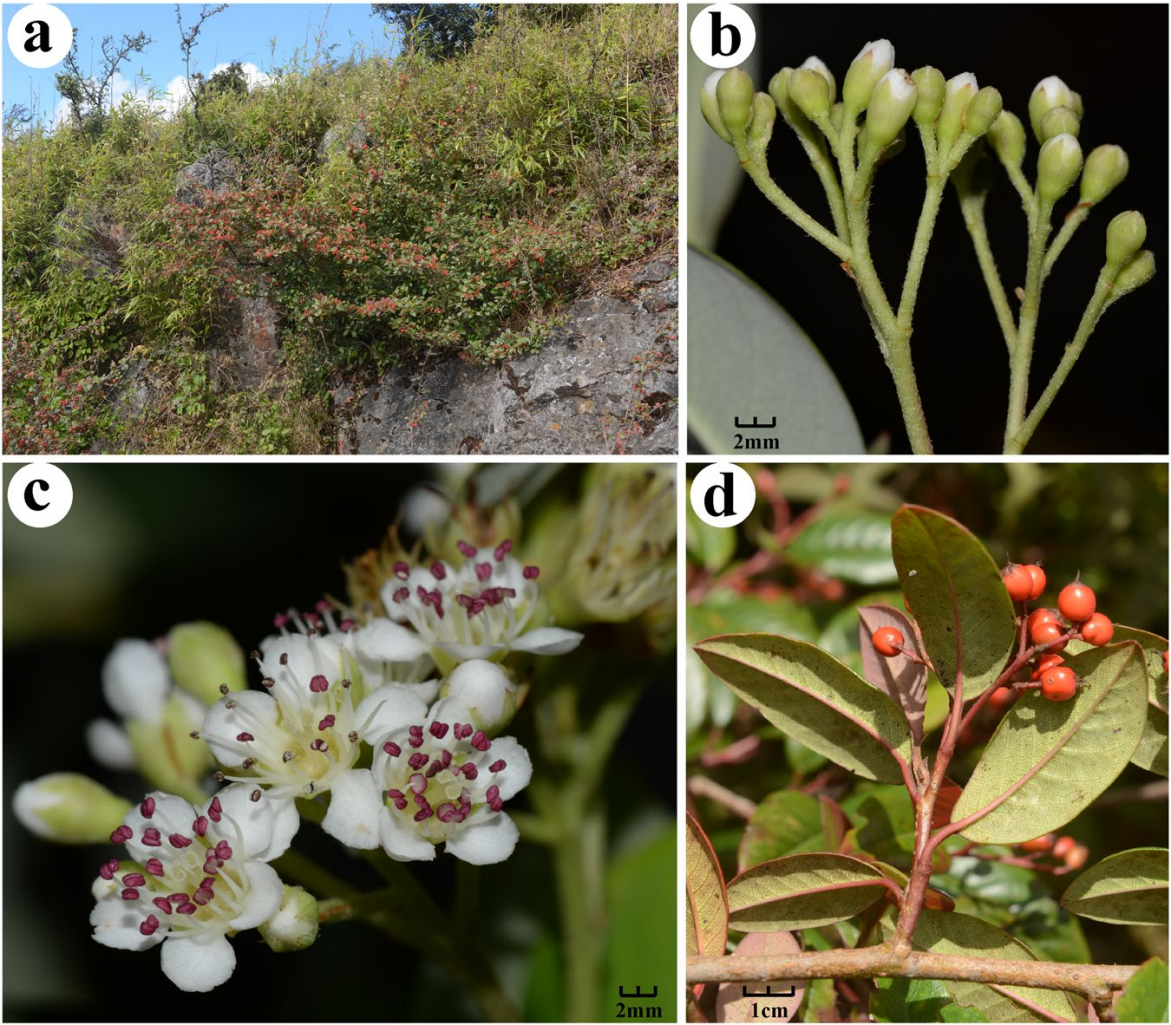
Photographs taken from the sampled plant (**a**–**d**) of *Cotoneaster glaucophyllus*. (**a**) habit; (**b**) inflorescences with floral buds; (**c**) bloomed flowers, showing white filaments and purple anthers; (**d**) mature fruits.

Using the Pacific Biosciences (PacBio) platform, we generated ~117 Gb of DNA continuous long reads (CLRs) and obtained ~48 Gb of full-length transcriptome sequences. Additionally, we sequenced ~104 Gb of DNA reads and ~10 Gb of RNA reads (2 × 150 bp) as well as ~62 Gb of high-throughput chromosome conformation capture (Hi-C) reads based on the Illumina HiSeq platform. With the aid of Hi-C technologies, we finally provided a high-quality genome sequence for the diploid species (2n = 2x = 34) of *C*. *glaucophyllus* (Fig. 1).

## Methods

### Sample collections

Fresh leaves, fruits and roots were collected from an adult plant of *C. glaucophyllus* (Xiajinchang, Malipo County, Yunnan Province, China; 23°08′26.57″N, 104°48′34.54″E; a.l.s. 1959 m; Fan17545, SYS!). The samples were separately wrapped in foil paper on 28 September, 2019 (Fig. 1a,d). Immediately thereafter, they were frozen in liquid nitrogen and then were preserved in Drikold and sent to Novogene Bioinformatics Technology Co., Ltd (Beijing, China). On 15 June, 2020, we collected flower tissue from the same plant (Specimen: Fan17951, SYS!) (Fig. 1b,c).

### DNA and RNA extraction and genome sequencing

Total DNA was extracted from fresh leaves using the Plant Genomic DNA Kit (DP305, Tiangen Biotech Co., Ltd., Beijing, China). The qualified DNAs were used to construct libraries intended for single molecular real-time (SMRT) sequencing using the Pacific Biosciences system (Menlo Park, CA, USA), Illumina sequencing, and Hi-C sequencing. The 20 kb library was prepared following the manufacturer’s protocol^13^. For the Illumina DNA paired-end library, the NEBNext® UltraTM DNA Library Prep Kit was utilized according to the provided instructions, with an insert size of 350 bp. The Hi-C library was prepared following standard procedures^14^.

Samples including fresh leaves, flowers, fruits, roots, and stems were pooled for total RNA extraction using the TIANGEN RNAPrep Pure Plant kit (DP432, Tiangen Biotech Co. Ltd., Beijing, China). Subsequently, the qualified RNAs were utilized for synthesizing full-length cDNAs with the SMRTer PCR cDNA Synthesis Kit (Biomarker, Beijing). Full-length transcriptome sequencing was performed on the PacBio Sequel platform. Additionally, short RNA-Seq reads (2 × 150 bp) specifically from leaf samples were generated and processed^15^ to facilitate the correction of the long-read RNA sequencing data and genome annotation.

PacBio long-read sequencing was performed using the PacBio Sequel system, while high throughput sequencing (2 × 150 bp) was carried out using an Illumina HiSeq sequencer. Both sequencing processes were conducted at Novogene Bioinformatics Technology Co., Ltd. (Beijing, China).

### Pre-estimation of genomic characteristics

**Fig. 2:**
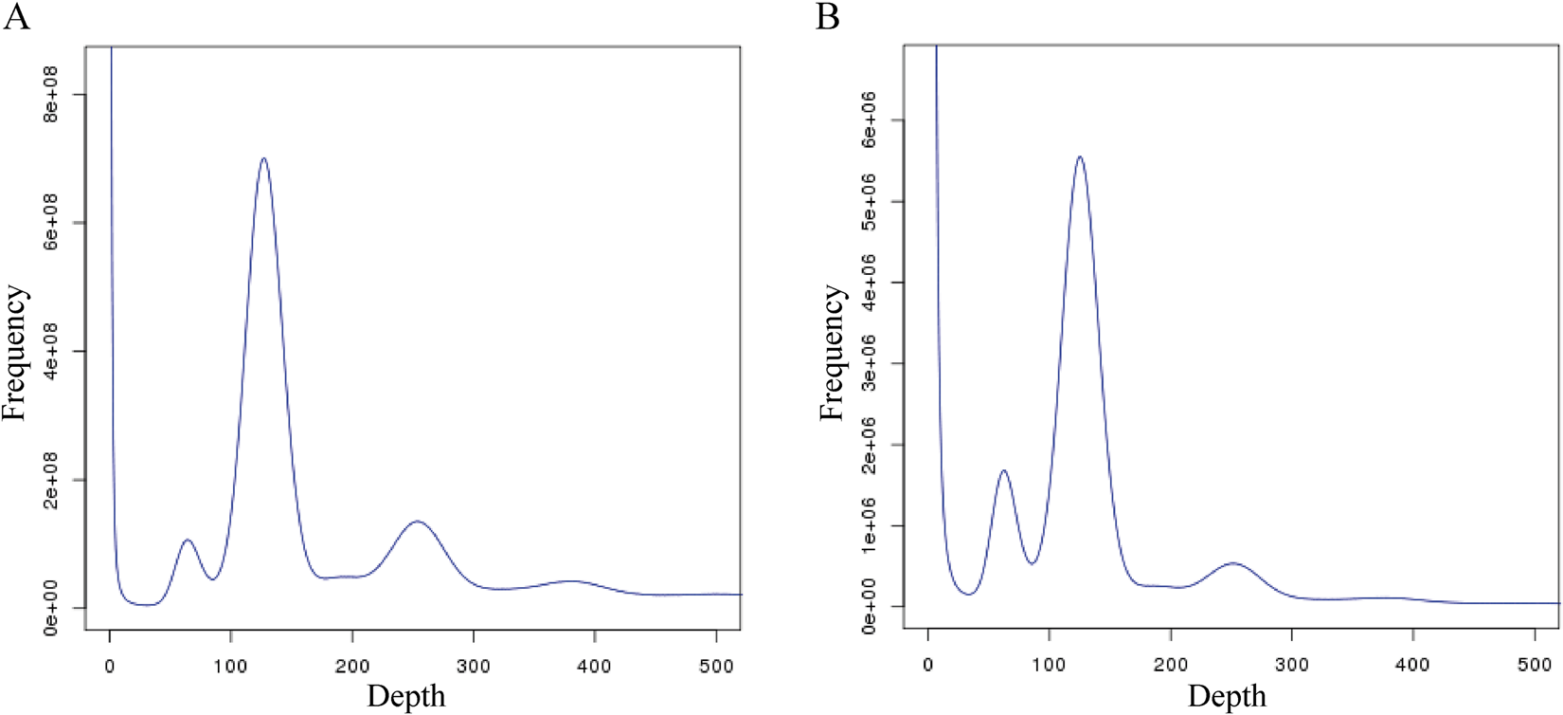
Frequency distribution of depth and K-mer numbers (**A**) and frequency distribution of depth and K-mer types (**B**).

The generated Illumina sequencing data were primarily processed using the NGSQC Toolkit v2.3.3^16^. This processing was involved in discarding reads that had adaptor contamination, reads with more than 10% unknown nucleotides (N), and paired reads that contained over 20% bases with a quality score of less than 5 in either read. Then, we performed a genome survey using Jellyfish v.2.2.7^17^ with the default setting of k-mer = 17 (Fig. 2). Based on a kmer-based statistical approach, GenomeScope v.2.0^18^ was used to estimate genome heterozygosity, repeat content, and size. To initially assess the genomic complexity, we employed SOAPdenovo v.2.0.4^19^ to generate a *de novo* draft assembly using a k-mer length of 41. The assembled contigs were then utilized to calculate the guanine-cytosine (GC) content. The estimated genome size was determined to be 625.87 Mb, with a heterozygosity rate of 0.55% and a repeat sequence proportion of 54.97%. Moreover, the estimated GC content was 38.65%.

### Genome assembly and quality assessment

The FALCON assembler^20^ was initially employed to perform self-correction of PacBio subreads. Subsequently, preassembled reads were assembled using the overlap-layout-consensus (OLC) algorithm, resulting in consensus contigs. To enhance the accuracy of the results, high-quality contigs were further corrected using Illumina short DNA reads through Pilon^21^. Leveraging the clean Hi-C data, the LACHESIS tool^22^ was utilized to scaffold the assembly, ultimately yielding a chromosome-level assembly. The *de novo* genome assembly was 563.3 Mb in length, with a contig N50 of ~6 Mb and a scaffold N50 of ~31 Mb (Table 1).

**Table 1:** Statistics of genome assembly.

Categories	Contig length (bp)	Scaffold length (bp)	Contig number	Scaffold number
Total	563,281,985	563,292,685	211	104
N50	6,038,690	30,996,934	31	8
N60	4,796,681	28,959,031	42	10
N70	3,810,158	28,326,837	55	12
N80	3,133,421	26,935,131	71	14
N90	1,900,000	25,186,462	93	16

**Fig. 3:**
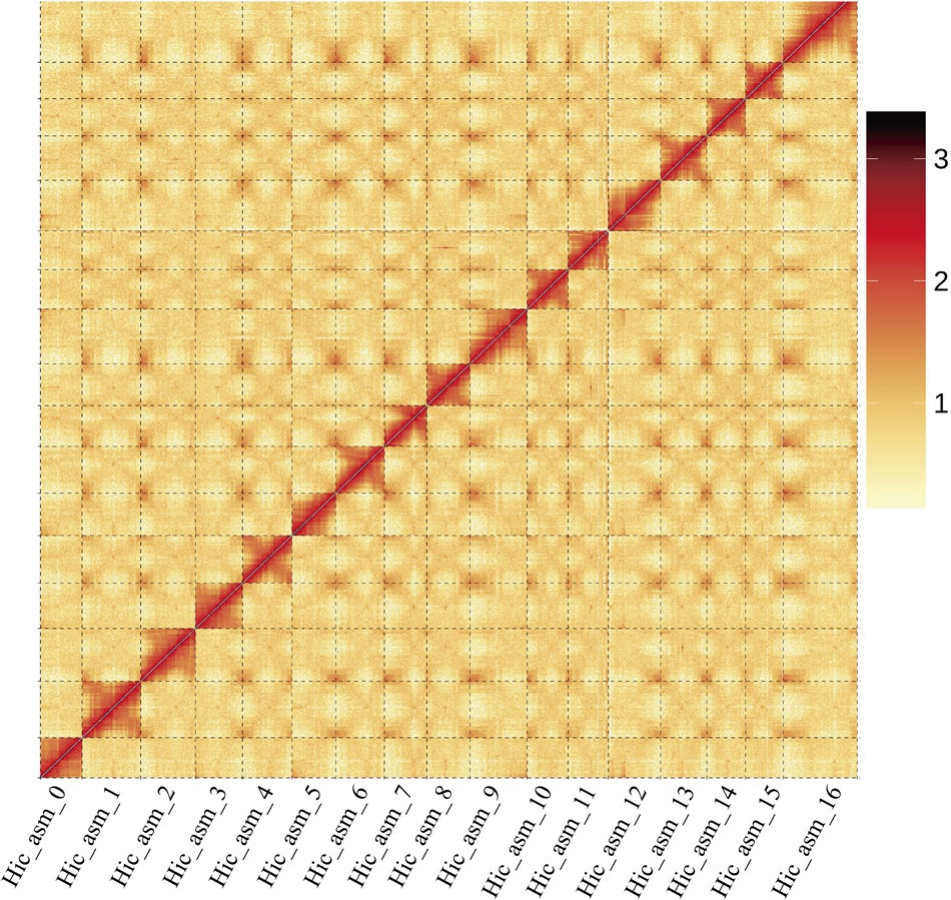
Hi-C interaction heatmap within pseudochromosomes of *Cotoneaster glaucophyllus*.

Among the 211 contigs, 124 were anchored to 17 pseudochromosomes (538.4 Mb, 95.59%) (Fig. 3, Table 2) and the remaining 87 were unanchored (24.9 Mb, 4.41%) (Table 2, Table S1). The GC content of these pseudochromosomes was ranging from 37.90% to 39.13% (Table 2).

**Table 2:** Summary of 17 pseudochromosomes and 87 contigs.

Pseudo-chromosome ID	Cluster number (contigs)	Sequences length (bp)	GC content (%)	Depth	Coverage
Hic_asm_0	3	27,504,221	39.13	178.463	100.0000%
Hic_asm_1	7	38,599,162	38.05	157.158	99.9999%
Hic_asm_2	8	36,284,958	38.12	164.872	100.0000%
Hic_asm_3	6	30,996,934	38.42	155.972	100.0000%
Hic_asm_4	9	32,331,078	37.94	162.567	99.9999%
Hic_asm_5	5	28,959,031	38.56	160.739	99.9995%
Hic_asm_6	7	31,856,070	38.14	161.755	99.9999%
Hic_asm_7	10	28,326,837	38.04	158.514	99.9996%
Hic_asm_8	3	28,498,786	38.13	163.358	100.0000%
Hic_asm_9	9	37,482,581	38.15	156.361	99.9997%
Hic_asm_10	4	26,935,131	37.90	160.833	100.0000%
Hic_asm_11	5	26,633,270	37.91	159.080	99.9999%
Hic_asm_12	7	34,559,293	38.44	163.268	99.9999%
Hic_asm_13	9	30,523,845	38.08	156.253	99.9998%
Hic_asm_14	9	25,186,462	38.25	162.008	99.9995%
Hic_asm_15	6	25,131,330	37.94	166.015	99.9983%
Hic_asm_16	17	48,616,628	38.21	158.499	99.9999%
Unplaced	87	24,867,068	38.26	161.989 (Average)	99.9998% (Average)
Total	124	538,425,617	38.20		

To comprehensively evaluate the reliability of the assembly, multiple assessments were performed in addition to considering the contig/scaffold N50 length. First, the integrity of the assembly was assessed by mapping the assembled genome to the BUSCO (Benchmarking Universal Single-Copy Orthologs) database v2.0^23^ (BUSCO, RRID: SCR 015008) and the CEGMA v2.5^24^ (Core Eukaryotic Genes Mapping Approach, RRID: SCR 015055). The BUSCO database contains 1,440 conserved core genes in terrestrial plants, while CEGMA includes a subset of the 248 most highly-conserved Core Eukaryotic Genes (CEGs). Second, the consistency between the assembly and paired-end Illumina short reads was evaluated by calculating the mapping and coverage rates. The Burrows‒Wheeler Aligner (BWA) v0.7.15^25^ was used to align the 150 bp short reads to the assembly. Thirdly, assembly accuracy was assessed by conducting SNP calling using SAMtools v1.9^26^ and BCFtools v1.9 (https://github.com/samtools/bcftools) based on the above mapping results. The rates of homozygous and heterozygous single-nucleotide polymorphisms (SNPs) were also determined.

### Genome annotation

We applied a combined strategy that utilized both *de novo* search and homology alignment to identify the repeats. A *de novo* repetitive element database was generated using LTR_FINDER v.1.0.6^27^, RepeatScout v.1.0.5^28^, Piler-DF v2.4^29^, and RepeatModeler v.2.0.1^30^ with the default parameters. The raw transposable element (TE) library included all repeat sequences that were longer than 100 bp and had less than 5% “N” gaps. To obtain a nonredundant library, a combined of Repbase^31^ and the raw TE library processing was conducted using uclust. Finally, RepeatMasker v.4.1.0^32^ was employed for the repeat identification using the nonredundant library. The homology-based approach utilized RepeatMasker v.4.1.0^32^ and the Repbase^31^ library to identify known transposable elements (TEs). These identified TEs were subsequently aligned with the genome sequences using a TE protein database, RepeatProteinMask v.4.1.0^32^. Tandem repeats were predicted using Tandem Repeats Finder v.4.09^33^. In the genome assembly, 55.60% repeat sequences were identified, among which 4.19% were tandem repeat sequences and 50.33% were long terminal repeat retrotransposons (LTR-RTs) (Table 3).

**Table 3:** Summary of interspersed repetitive sequences.

Categories	Repeatmasker		Proteinmask		Combined TEs	
	Length (bp)	Proportion(%)	Length (bp)	Proportion(%)	Length (bp)	Proportion(%)
DNA	10,928,552	1.94	2,122,128	0.38	11,963,490	2.12
LINE	2,410,920	0.43	4,590,916	0.82	5,767,793	1.02
SINE	42,316	0.01	0	0	42,316	0.01
LTR	280,509,306	49.8	66,260,707	11.76	283,507,978	50.33
Unknown	11,275,248	2	0	0	11,275,248	2
Total	302,404,476	53.69	72,971,214	12.95	306,550,288	54.42

Multiple approaches, including *ab initio* prediction, homology-based prediction, and full-length transcript evidence, were employed to annotate gene models. For *ab initio* gene predication based on *ab initio*, GeneWise v.2.4.1^34^, Augustus v3.2.3^35^, Geneid v1.4^36^, Genescan v3.1^37^, GlimmerHMM v3.04^38^, and SNAP^39^ were used. Homologous protein sequences of *Malus* x *domestica*^40^, *Fragaria vesca*^41^, *Rosa chinensis*^42^, *Prunus persica*^43^, *Pyrus betuleafolia*^44^, and *Eriobotrya japonica*^12^ were downloaded from NCBI (https://www.ncbi.nlm.nih.gov/genome/) and then were aligned to the assembly using tBLASTn v2.2.26^45^ (E-value ≤ 1e-5). The matching proteins were aligned to the homologous genome sequences for accurate spliced alignments with GeneWise v2.4.1^34^ software. The IsoSeq pipeline (https://github.com/PacificBiosciences/IsoSeq) was employed to process full-length transcriptome sequencing data. The generated reads were aligned to *C. glaucophyllus* using HISAT v.2.0.4^46^ with the default parameters and then the alignment was further processed by StringTie v.1.3.3^47^. The nonredundant reference gene set was created by merging the genes predicted as described above with EVidenceModeler v1.1.1^48^ using PASA^49^ (Program to Assemble Spliced Alignment) terminal exon support and including masked transposable elements as aninput for gene prediction. Furthermore, gene structure and gene elements, including average transcript length, average CDS length, and average exon and intron length, were compared among *Cotoneaster glaucophyllus* and the above six related species.

The tRNAs were predicted using the tRNAscan-SE^50^ program (http://lowelab.ucsc.edu/tRNAscan-SE/). As rRNAs are highly conserved, we selected reference rRNA sequences from closely related species and used BLAST to predict rRNA sequences. Additionally, other ncRNAs, such as miRNAs and snRNAs, were identified by searching against the Rfam^51^ database using the Infernal v1.1^34^ with the default parameters. We annotated 35,856 coding genes (Tables 4) and 3,276 noncoding genes, including 1,401 miRNAs, 655 tRNAs, 425 rRNAs, and 795 snRNAs (Table 5).

**Table 4:** Statistics of gene structure prediction.

Methods	Gene set	Gene number	Average length of transcript (bp)	Average length of CDS (bp)	Average number of exon (bp)	Average length of exon (bp)	Average length of intron (bp)
*Ab initio* annotation	Augustus	37,027	2,452.43	1,101.80	4.59	239.95	376.03
	GlimmerHMM	62,020	7,100.45	721.87	3.19	226.08	2,908.63
	SNAP	41,045	4,788.95	680.84	4.2	162.13	1,284.05
	Geneid	60,430	4,120.01	800.58	4.11	194.94	1,068.45
	Genscan	38,351	9,234.19	1,250.75	6.17	202.74	1,544.46
Homologous annotation	*Malus* x *domestica*	35,332	2,467.52	1,022.76	4.53	225.98	409.75
	*Eriobotrya japonica*	33,185	2,622.72	1,097.65	4.77	230.29	404.91
	*Prunus persica*	30,757	2,603.98	1,109.13	4.74	234.13	399.98
	*Rosa chinensis*	30,612	2,637.62	1,102.39	4.7	234.5	414.8
	*Fragaria vesca*	26,440	3,177.46	1,173.02	5.02	233.5	498.16
	*Pyrus betuleafolia*	34,833	2,701.08	1,030.36	4.6	223.89	463.83
Transcriptome annotation	PASA	51,317	2,960.31	1,088.83	5.14	211.75	451.81
	Transcripts	29,081	6,337.00	1,959.58	6.48	302.46	798.96
EVM		42,425	2,907.91	1,043.29	4.56	228.83	523.89
PASA-update*		42,285	2,887.94	1,053.94	4.58	230.36	512.98
Final set*		35,856	3,195.06	1,152.68	5.02	229.78	508.51

Note: The asterisk (*) indicates the inclusion of UTR regions.

**Table 5:** Statistics of noncoding genes.

Categories	Number	Average length (bp)	Total length (bp)	Proportion (%)
**miRNA**	1,401	170.7	239,157	0.042457
**tRNA**	655	75.05	49,157	0.008727
**rRNA**	425	155.03	65,886	0.011697
18 S	28	779.86	21,836	0.003876
28 S	50	132.62	6,631	0.001177
5.8 S	11	146.09	1,607	0.000285
5 S	336	106.58	35,812	0.006358
**snRNA**	795	119.83	95,267	0.016913
CD-box	429	105.41	45,221	0.008028
HACA-box	64	130.08	8,325	0.001478
splicing	297	137.82	40,932	0.007267
scaRNA	5	157.8	789	0.00014

**Fig. 4:**
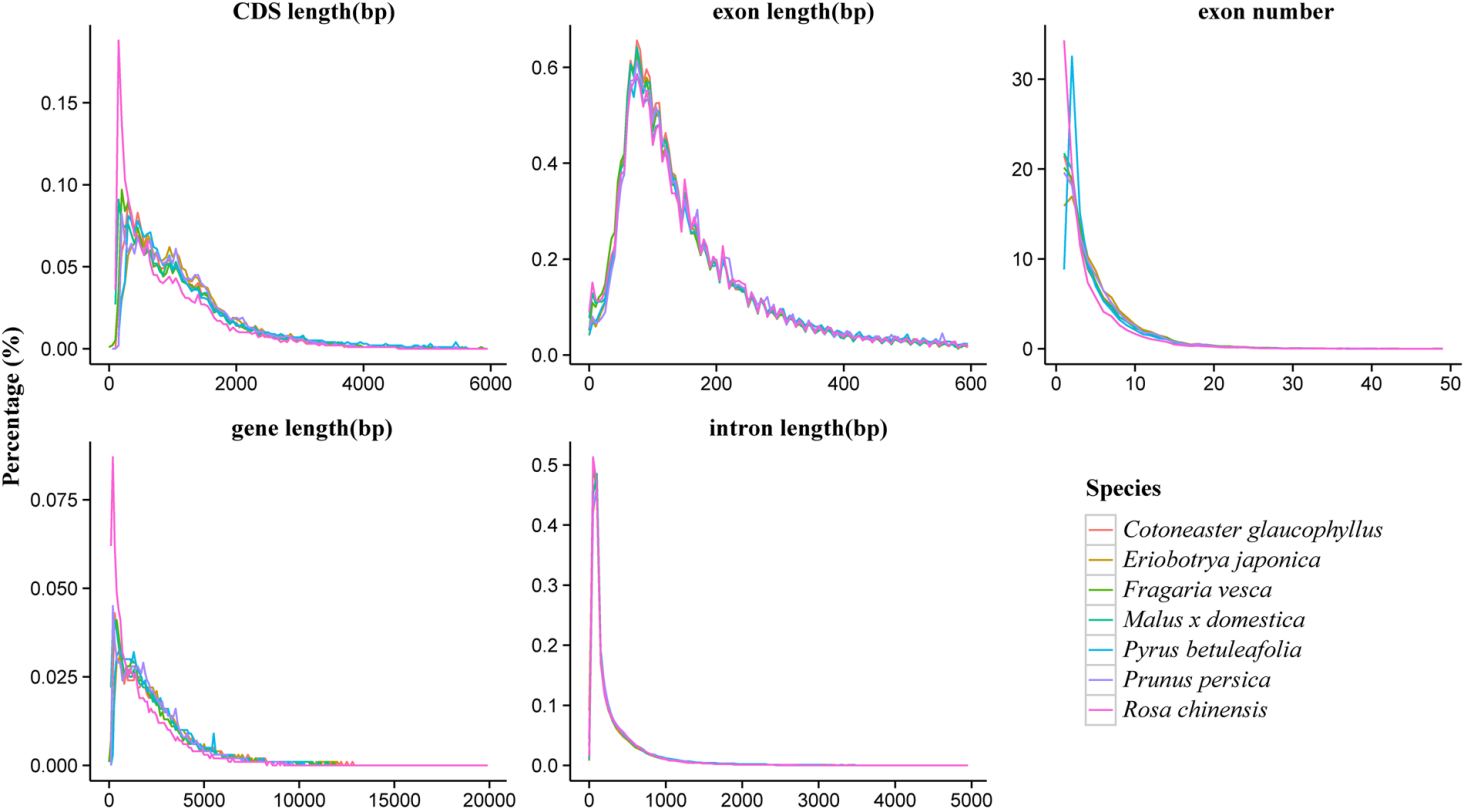
Comparative analysis of gene elements among Rosaceae-related species.

Gene functions were assigned by aligning the protein sequences to Swiss-Prot^52^ using Blastp^53^, with a threshold of E-value ≤ 1e−5, and the best match was considered. Motifs and domains were annotated using InterProScan v5.31^54^, which involved searching against publicly available databases, including ProDom^55^, PRINTS^56^, Pfam^57^, SMART^58^, PANTHER^59^, and PROSITE^60^. Gene Ontology (GO) IDs were assigned to each gene based on the corresponding InterPro entry. Protein function predictions were made by transferring annotations from the closest BLAST hit (E-value ≤ 1e−5) in the SwissProt database^51^ and DIAMOND v0.8.22^61^ hit (E-value ≤ 1e−5) in the NR database. Additionally, we mapped the gene set to a KEGG pathway and identified the best match for each gene. The functions of 34,967 genes (97.52%) were predicted (Table 6). Comparative analysis of gene elements among Rosaceae-related species revealed that the genome assembly of *Cotoneaster glaucophyllus* exhibits a shorter average exon length (229.78 bp) and a longer average intron length (508.51 bp) than those of other considered species (Fig. 4, Table 7).

**Table 6:** Summary of gene function annotations.

Categories	Annotated gene number	Percent (%)
Total	35,856	—
Swissprot	27,031	75.40
Nr	34,880	97.30
KEGG	27,206	75.90
InterPro	32,245	89.90
GO	19,267	53.70
Pfam	26,399	73.60
Annotated	34,967	97.50
Unannotated	889	2.50

**Table 7:** Comparative analysis of gene elements among Rosaceae-related species.

Species	Number	Average transcript length (bp)	Average CDS length (bp)	Average exons per gene	Average exon length (bp)	Average intron length (bp)
*Cotoneaster glaucophyllus*	35856	3,195	1,152.68	5.02	229.78	508.51
*Pyrus betuleafolia*	59552	2,801	1,305.11	4.73	275.86	401.06
*Fragaria vesca*	28588	2,571	1,177.72	4.98	236.46	349.95
*Prunus persica*	28705	2,468	1,211.02	4.97	243.58	316.48
*Rosa chinensis*	45469	1,905	961.61	3.83	251.15	333.53
*Malus* x *domestica*	45116	2,543	1,127.16	4.78	235.58	374.13
*Eriobotrya japonica*	45743	3,083	1,262.13	5.28	239.2	425.71

## Data Records

The raw data of Hi-C short reads, Illumina DNA short reads, PacBio DNA long reads, RNA short reads, and PacBio RNA long reads have been deposited in the National Center for Biotechnology Information (NCBI) Sequence Read Archive database with accession numbers SRR25933879^62^, SRR25933878^63^, SRR25933877^64^, SRR25933876^65^, and SRR25933875^66^ under BioProject accession number PRJNA1012579. The genome assembly has been deposited at GenBank under the WGS accession JAVVNS000000000^67^. Additionally, the genome assembly, predicted transcripts and protein sequences, functional annotation files (gff files), and NR and KEGG annotation files have been deposited in Figshare^68^.

## Technical Validation

Multiple parameters were employed to assess the quality of the genome assembly. The BUSCO evaluation indicated that among the Eukaryota BUSCO genes, 62.9% (906) of the sequences were identified as complete and single-copy, while 30.3% (436) were complete but duplicated. Additionally, 1.1% (16) of the sequences were fragmented, and 5.7% (82) were found to be missing. Analysis of the 248 most highly-conserved Core Eukaryotic Genes (CEGs) revealed the presence of 238 complete genes (95.97%) and 6 incomplete genes (2.42%). The evaluation of the consistency between the assembly and paired-end DNA short reads indicated that the overall mapping and coverage rates were 94.61% and 99.99%, respectively. The rates of homozygous and heterozygous single-nucleotide polymorphisms (SNPs) were 0.001413% (798) and 0.288695% (163,081). Furthermore, we mapped the DNA continuous long reads (CLRs) to the genome using the minimap2^69^, and calculated the sequencing depth and coverage for each pseudo-chromosome (Table 2). These results collectively demonstrate a genome assembly of high quality, completeness, and accuracy.

### Supplementary information

Table S1

## Data Availability

All software and pipelines were executed in strict accordance with the manuals and protocols provided by the published bioinformatic tools. No custom programming or coding was used.

## References

[1] The Angiosperm Phylogeny Group (2016). An update of the Angiosperm Phylogeny Group classification for the orders and families of flowering plants: APG IV. Botanical Journal of the Linnean Society.

[2] Fryer, J. & Hylmö, B. *Cotoneasters: A Comprehensive Guide To Shrubs for Flowers, Fruit, and Foliage*. (Timber Press, Portland and London, 2009).

[3] Meng KK (2021). Phylogenomic analyses based on genome-skimming data reveal cyto-nuclear discordance in the evolutionary history of *Cotoneaster* (Rosaceae). Mol Phylogenet Evol.

[4] Robertson KR (1991). A synopsis of genera in Maloideae (Rosaceae). Syst Bot.

[5] Li FF (2014). Molecular phylogeny of *Cotoneaster* (Rosaceae) inferred from nuclear ITS and multiple chloroplast sequences. PLANT Syst Evol.

[6] Lu, L. D. *et al*. Rosaceae. In Wu, Z.Y. and Raven, P.H. (Eds.). Flora of China. Science Press, Beijing, China and Missouri Botanical Garden Press, St. Louis. **9**, 46–434 (2003).

[7] Yü, T. T. *et al*. Rosaceae. In: Yü, T. T. (Ed.), Flora Reipublicae Popularis Sinicae. Science Press, Beijing **36**, 107–178 (1974).

[8] Cao K (2022). Chromosome-level genome assemblies of four wildpeach species provide insights into genome evolution and genetic basis of stress resistance. BMC Biol.

[9] Soyturk A (2021). De novo assembly and characterization of the first draft genome of quince (*Cydonia oblonga* Mill.). Sci Rep.

[10] Zhang J (2020). The high-quality genome of diploid strawberry (*Fragaria nilgerrensis*) provides new insights into anthocyanin accumulation. Plant Biotechnol J.

[11] Sun X (2020). Phased diploid genome assemblies and pan-genomes provide insights into the genetic history of apple domestication. Nat Genet.

[12] Jiang, S. *et al*. Chromosome-level genome assembly and annotation of the loquat (*Eriobotrya japonica*) genome. *Gigascience* **9** (2020). 10.1093/gigascience/giaa015 PMC7059265 32141509

[13] Guidelines for Preparing 20 kb SMRTbell TM Templates, https://www.pacb.com/wp-content/uploads/2015/09/User-Bulletin-Guidelines-for-Preparing-20-kb-SMRTbell-Templates.pdf Accessed on 25 Nov 2020.

[14] Belton JM (2012). Hi-C: a comprehensive technique to capture the conformation of genomes. Methods.

[15] Meng, K. K. *et al*. Isolation and identification of EST-SSR markers in *Chunia bucklandioides* (Hamamelidaceae). *Appl Plant Sci* **4** (2016). 10.3732/apps.1600064 PMC5077281 27785382

[16] Patel RK, Jain M (2012). NGS QC Toolkit: A Toolkit for Quality Control of Next Generation Sequencing Data. PLOS ONE.

[17] Marcais G, Kingsford C (2011). A fast, lock-free approach for efficient parallel counting of occurrences of k-mers. Bioinformatics.

[18] Ranallo-Benavidez TR (2020). GenomeScope 2.0 and Smudgeplot for reference-free profiling of polyploid genomes. Nat Commun.

[19] Luo R (2012). SOAPdenovo2: an empirically improved memory-efficient short-read de novo assembler. Gigascience.

[20] Chin CS (2016). Phased diploid genome assembly with single-molecule real-time sequencing. Nat Methods.

[21] Walker BJ (2014). Pilon: an integrated tool for comprehensive microbial variant detection and genome assembly improvement. PLoS One.

[22] Sedayao, J. & Akita, K. LACHESIS: A Tool for Benchmarking Internet Service Providers (1995).

[23] Simao FA (2015). BUSCO: assessing genome assembly and annotation completeness with single-copy orthologs. Bioinformatics.

[24] Parra G (2007). CEGMA: a pipeline to accurately annotate core genes in eukaryotic genomes. Bioinformatics.

[25] Li H, Durbin R (2009). Fast and accurate short read alignment with Burrows-Wheeler transform. Bioinformatics.

[26] Li H (2009). The Sequence Alignment/Map format and SAMtools. Bioinformatics.

[27] Xu Z, Wang H (2007). LTR_FINDER: an efficient tool for the prediction of full-length LTR retrotransposons. Nucleic Acids Res.

[28] Price AL (2005). De novo identification of repeat families in large genomes. Bioinformatics.

[29] Edgar RC, Myers EW (2005). PILER: identification and classification of genomic repeats. Bioinformatics.

[30] Flynn JM (2020). RepeatModeler2 for automated genomic discovery of transposable element families. Proc Natl Acad Sci USA.

[31] Bao W (2015). Repbase Update, a database of repetitive elements in eukaryotic genomes. Mob DNA.

[32] Tarailo-Graovac, M. & Chen, N. Using RepeatMasker to identify repetitive elements in genomic sequences. *Curr Protoc Bioinformatics* Chapter 4, 4.10 (2009). 10.1002/0471250953.bi0410s25 19274634

[33] Benson G (1999). Tandem repeats finder: a program to analyze DNA sequences. Nucleic Acids Res.

[34] Nawrocki EP, Eddy SR (2013). Infernal 1.1: 100-fold faster RNA homology searches. Bioinformatics.

[35] Stanke M (2004). AUGUSTUS: a web server for gene finding in eukaryotes. Nucleic Acids Res.

[36] Alioto T (2018). Using geneid to Identify Genes. Curr Protoc Bioinformatics.

[37] Burge C, Karlin S (1997). Prediction of complete gene structures in human genomic DNA. J Mol Biol.

[38] Majoros WH (2004). TigrScan and GlimmerHMM: two open source ab initio eukaryotic gene-finders. Bioinformatics.

[39] Bromberg Y, Rost B (2007). SNAP: predict effect of non-synonymous polymorphisms on function. Nucleic Acids Res.

[40] Zhang L (2019). A high-quality apple genome assembly reveals the association of a retrotransposon and red fruit colour. Nat Commun.

[41] Shulaev V (2011). The genome of woodland strawberry (*Fragaria vesca*). Nat Genet.

[42] Raymond O (2018). The *Rosa* genome provides new insights into the domestication of modern roses. Nature Genetics.

[43] Lian X (2022). De novo chromosome-level genome of a semi-dwarf cultivar of *Prunus persica* identifies the aquaporin PpTIP2 as responsible for temperature-sensitive semi-dwarf trait and PpB3-1 for flower type and size. Plant Biotechnol J.

[44] Dong X (2020). De novo assembly of a wild pear (*Pyrus betuleafolia*) genome. Plant Biotechnol J.

[45] NCBI. BLASTALL v2.2.26. Bethesda, MD: National Center for Biotechnology Information. (2009).

[46] Kim D (2015). HISAT: a fast spliced aligner with low memory requirements. Nat Methods.

[47] Pertea M (2016). Transcript-level expression analysis of RNA-seq experiments with HISAT, StringTie and Ballgown. Nat Protoc.

[48] Haas BJ (2008). Automated eukaryotic gene structure annotation using EVidenceModeler and the Program to Assemble Spliced Alignments. Genome Biol.

[49] Haas BJ (2003). Improving the *Arabidopsis* genome annotation using maximal transcript alignment assemblies. Nucleic Acids Res.

[50] Lowe TM, Eddy SR (1997). tRNAscan-SE: a program for improved detection of transfer RNA genes in genomic sequence. Nucleic Acids Res.

[51] Griffiths-Jones S (2005). Rfam: annotating non-coding RNAs in complete genomes. Nucleic Acids Res.

[52] Bairoch A, Apweiler R (2000). The SWISS-PROT protein sequence database and its supplement TrEMBL in 2000. Nucleic Acids Res.

[53] Gish W, States DJ (1993). Identification of protein coding regions by database similarity search. Nat Genet.

[54] Jones P (2014). InterProScan 5: genome-scale protein function classification. Bioinformatics.

[55] Gouzy J (1997). XDOM, a graphical tool to analyse domain arrangements in any set of protein sequences. Comput Appl Biosci.

[56] Attwood TK (2012). The PRINTS database: a fine-grained protein sequence annotation and analysis resource–its status in 2012. Database (Oxford).

[57] El-Gebali S (2019). The Pfam protein families database in 2019. Nucleic Acids Res.

[58] Letunic I (2004). SMART 4.0: towards genomic data integration. Nucleic Acids Res.

[59] Mi H. Y (2013). PANTHER in 2013: modeling the evolution of gene function, and other gene attributes, in the context of phylogenetic trees. Nucleic Acids Res.

[60] Sigrist CJA (2012). New and continuing developments at PROSITE. Nucleic Acids Research.

[61] Buchfink B (2015). Fast and sensitive protein alignment using DIAMOND. Nat Methods.

[62] (2023). NCBI Sequence Read Archive.

[63] (2023). NCBI Sequence Read Archive.

[64] (2023). NCBI Sequence Read Archive.

[65] (2023). NCBI Sequence Read Archive.

[66] (2023). NCBI Sequence Read Archive.

[67] Meng K. K (2024). GenBank.

[68] Meng K. K (2023). Figshare.

[69] Li H (2018). Minimap2: pairwise alignment for nucleotide sequences. Bioinformatics.

